# Observing in real time the evolution of artemisinin resistance in *Plasmodium falciparum*

**DOI:** 10.1186/s12916-015-0316-5

**Published:** 2015-03-31

**Authors:** Carol Hopkins Sibley

**Affiliations:** Worldwide Antimalarial Resistance Network, Department of Genome Sciences, University of Washington, Seattle, WA USA

**Keywords:** Malaria, *Plasmodium falciparum*, Artemisinin Resistance, Molecular Markers, Genomic Analysis

## Abstract

Simple genetic changes that correlate with drug resistance are used routinely to identify resistant pathogens. These “molecular markers” have usually been defined long after the phenotype of resistance was noted. The molecular changes at the “end game” reflect a long and complex evolution of genetic changes, but once a solidly resistant set of changes assembles under drug selection, that genotype is likely to become fixed, and resistant pathogens will spread widely.

Artemisinins are currently used worldwide to treat malaria caused by *Plasmodium falciparum*, but parasite response has diminished rapidly in the Mekong region of Southeast Asia. Should artemisinins lose potency completely and this effect spread worldwide, effective malaria treatment would be almost impossible. The full range of modern methods has been applied to define rapidly the genetic changes responsible. Changes associated with artemisinin resistance are complex and seem to be evolving rapidly, especially in Southeast Asia. This is a rare chance to observe the early stages in evolution of resistance, and to develop strategies to reverse or mitigate the trend and to protect these key medicines.

## Background

There may be no better scientific place to study an evolutionary arms race than the interaction between pathogens and the drugs needed to treat them. The clear importance to public health, the relatively rapid time frame of pathogen responses, and the richness of the data available provide both context and motivation for these studies [1]. The interaction of *Plasmodium falciparum*, the parasite that causes the most dangerous form of malaria, with antimalarial drugs provides an outstanding example. In 2008, it was first noted that *P. falciparum* in western Cambodia were evolving resistance to the artemisinin drugs, the cornerstone of current global antimalarial policy [2-4]. A paper published recently in *Nature Genetics* provides new insights into the genetic complexity and population structure of *P. falciparum* in an area where the resistance is evolving rapidly [5].

Artemisinins are one principal component of artemisinin combination therapies (ACTs). In the combination, the artemisinin component clears the parasite rapidly from the patient’s blood, but depends upon a long-acting partner drug to dispatch the remaining parasite biomass. One of the earliest indications that the artemisinin drugs are compromised is a delay in the initial clearance of the parasites in the first days after treatment [2,3]. The rate of parasite clearance is a challenging metric to quantify, but further studies were then launched to determine the extent of the resistant phenotype in the Mekong region [6-11] and Africa [12]. A collaborative effort by the research community developed a practical tool to assess the rate of parasite clearance reproducibly between studies [13], and a laboratory-based assay that correlates with the slow clearance phenotype was developed and validated [14-16]. In 2011 an extensive multicenter trial of artemisinin efficacy in 13 sites in the Greater Mekong region and 2 in Africa was launched, the Tracking Resistance to Artemisinin Collaboration (TRAC) [17].

## Molecular markers of resistance

Previous studies defined molecular markers for resistance to antimalarials long after the resistant parasites were widespread [18], but this time, modern genomic and analytical tools were available, and identification of a simple molecular marker to track the slow-clearing parasites was an immediate goal. The phenotype was shown to depend largely on the genotype of the parasite [19], and combining data from the field studies, slow-clearing parasites from Cambodia [10,20] and western Thailand [21] were shown to have some broad genomic regions in common. In late 2014, a molecular signature in what is called the “propeller region” of the highly conserved *Kelch 13* gene (K13, PF3D7_1343700), was shown to correlate strongly with the slow-clearing phenotype in Cambodian parasites [22]. Rapidly, the K13 genotypes of parasites from the TRAC study and earlier field studies in the Southeast Asian region were determined [17,23,24], and other studies are being added rapidly [25]. More than 30 different K13 mutant alleles have been identified; each carries a single nucleotide polymorphism (SNP) that changes one amino acid in the propeller region of the gene. So far in the Mekong region, most of the parasites with these mutant alleles correlate with slow clearance [17,22-24].

A few of these mutant alleles are very common, observed in several locations, but others are rare, identified in a single location or parasite population. When the DNA flanking the coding region of K13 was examined, two very common alleles had spread locally, but these same K13 alleles had also emerged independently on different genetic backgrounds [23]. Miotto and colleagues determined full genome sequences of parasites collected in the TRAC study and compared the genetic signatures of slow- and fast-clearing parasites [5]. As expected, the slow-clearing parasites carried a variety of K13 mutant alleles; analysis of the extended haplotypes surrounding the various K13 alleles confirmed that both common and rare alleles have arisen recently and independently in many different parasite populations.

Miotto and colleagues employed a genome-wide association study (GWAS) to identify genes associated with the slow clearance phenotype. This approach verified that the presence of any K13 propeller mutant allele was by far the strongest signal associated with slow clearance, as had been noted earlier [23]. However, they also identified four other loci on three different chromosomes that showed very strong association; all had prior connections with antimalarial resistance [*fd* (ferredoxin), *arps10* (apicoplast ribosomal protein S10), *mdr2* (multidrug resistance protein 2), and *crt* (chloroquine resistance transporter)]. When the carriage of “any K13 allele” was included as a covariate in the GWAS, these other loci made only modest contributions to the association, so this correlation with slow clearance largely reflects their population-based relationship with K13 alleles, not a functional role in artemisinin resistance.

## Genetic complexity and artemisinin use

In Cambodia and many other locations, artemisinins have been used and misused over an extended period, and malaria transmission has declined rapidly since 2008. Under these circumstances, it is not surprising that some of these parasite populations showed a reduced genetic complexity characteristic of founder populations [20]. To understand this observation more clearly, the authors defined seven founder populations that each carried a prominent K13 mutant allele. Despite their overall genetic difference, these populations frequently shared the same alleles of the *fd*, *arps10*, *mdr2*, and *crt* loci originally identified in the GWAS of all parasites under study. Analysis of these genetic relationships demonstrated that the auxiliary alleles must have evolved in a population ancestral to the founder populations, and the different K13 mutant alleles were selected later in each founder population, but on this common genetic background. Viewed in this light, the auxiliary alleles constitute a “permissive genome” common among parasites in the Mekong basin, a foundation on which K13 mutants not only occur, but survive, and often rise to high prevalence under selection by artemisinins.

This work from the Greater Mekong region makes it clear that the evolution of resistance to artemisinin is still evolving rapidly. Molecular surveys of the prevalence of K13 mutant alleles in Africa and India also suggest that the changes are at a very early stage there. The propeller region of K13 is highly conserved [26,27], and slow parasite clearance has not been observed in either area [12,17,28,29], so mutations in the propeller of the K13 gene were expected to be rare, or absent. Instead, K13 mutants were identified at low prevalence (<5%) in almost all African locations examined [28,30-32]; 25 K13 mutant alleles were identified, 21 that were novel and 4 that had been identified previously in the Mekong. Four sites in India each had a single isolate carrying a different K13 mutant allele; and two of these were novel [29].

More than 50 different K13 mutant alleles have now been identified, and many more are being reported as geographic surveys are widened. In the Mekong region, the correlation of K13 mutants with slow clearance is strong, and appears to depend upon the presence of the permissive genomes identified by Miotto and colleagues. In other parts of the world, it is not yet known whether the presence of a K13 mutant allele alone is enough to identify slow-clearing parasites. New tools may help to answer this important question. For example, when artemisinin-sensitive parasites were engineered to express a common K13 mutant allele from Cambodia, they were protected against artemisinin exposure in the laboratory [33,34]; testing the African or Indian alleles in this system could be a first step in determining the importance of these novel alleles to the parasite response to artemisinins. In addition, earlier studies have shown that artemisinin treatment increases oxidative stresses in the parasites [35] and slow-clearing parasites survive the treatment by retarding their progress through the life cycle and activating transcription of several families of genes that protect against these stresses [36,37]. These transcriptional changes are likely to be another manifestation of the “permissive genome” observed in the Mekong parasites. Other genetic changes may be required to support the emergence of K13 mutants in new selective environments, but sets of genes that are adapted to those locations could be identified using the approach described by Miotto and colleagues.

All of these studies have involved collaborations among a very large international group of researchers, combining expertise in clinical management, epidemiology, and genomics, and these studies provide an expanding understanding of the mechanism of resistance to the artemisinins. In the past, molecular markers of resistance have been identified only at the culmination of a long selection process, and a few alleles sufficed to identify resistant parasite populations over wide geographic areas [38-42]. At this stage, it is still too early to be sure whether the K13 molecular signature can be used alone as a valid marker for the slow clearance phenotype worldwide. A few parasite populations carrying common K13 alleles may prove to be best adapted to the selection pressures, increase, and slowly spread into neighboring areas, while most parasites with rare alleles may be transitory, disappearing in the evolutionary struggle [43]. Whatever the outcome, the tools are being assembled to answer that important question as quickly as possible.

## Conclusions

From the perspective of public health, patients treated with an ACT still recover in most of the world, so complete failure of ACT treatment is not as widespread as the K13 genotypes in the Mekong region and certainly not in other areas. However, with continued artemisinin pressure, other genetic changes may evolve and render these parasites even more artemisinin resistant. Even more worrying, the efficacy of ACTs requires both components, and when artemisinin loses potency, the partner drug is exposed to far greater selective pressure. When that happens, clinical efficacy of the ACT can diminish rapidly [9]. To short circuit this threat, elimination of *P. falciparum* parasites in the Mekong region must be a top priority. These new insights on the evolution of artemisinin resistance must also be turned into practical tools to detect artemisinin-resistant parasites in all areas, so that public health measures can be mobilized to contain their emergence or spread long before the clinical efficacy of ACTs is exhausted.

## Abbreviations

ACTs: artemisinin combination therapies
*arps10*: apicoplast ribosomal protein S10 gene, PF3D7_1460900.1
*crt*: chloroquine resistance transporter gene, PF3D7_0709000
*fd*: ferredoxin gene, PF3D7_1318100
GWAS: genome-wide association study
K13: *Kelch 13* gene, PF3D7_1343700
*mdr2*: multidrug resistance protein 2 gene, PF3D7_1447900
SNP: single nucleotide polymorphism
TRAC: Tracking Resistance to Artemisinin Collaboration
